# From forensic epigenetics to forensic epigenomics: broadening DNA investigative intelligence

**DOI:** 10.1186/s13059-017-1373-1

**Published:** 2017-12-21

**Authors:** Athina Vidaki, Manfred Kayser

**Affiliations:** Department of Genetic Identification, Erasmus MC University Medical Center Rotterdam, Room Ee1051, PO Box 2040, 3000 CA Rotterdam, The Netherlands

## Abstract

Human genetic variation is a major resource in forensics, but does not allow all forensically relevant questions to be answered. Some questions may instead be addressable via epigenomics, as the epigenome acts as an interphase between the fixed genome and the dynamic environment. We envision future forensic applications of DNA methylation analysis that will broaden DNA-based forensic intelligence. Together with genetic prediction of appearance and biogeographic ancestry, epigenomic lifestyle prediction is expected to increase the ability of police to find unknown perpetrators of crime who are not identifiable using current forensic DNA profiling.

## Introduction

Human genetic variation provides high discriminatory power in identifying known persons, such as perpetrators of crime [1, 2]. Although less established, it can also aid in predicting appearance traits and biogeographic ancestry, which is useful for finding unknown persons who are not identifiable with standard DNA profiling [3, 4]. While the genome is typically non-informative regarding lifelong environmental influences on the body, which can provide forensically relevant information, the epigenome acts as an interphase between the mostly “fixed” genome and the principally “dynamic” environment [5]. For example, lifelong molecular responses to environmental exposure via varying DNA methylation levels at thousands of cytosines across the genome result in individual epigenome variation [6–10].

In contrast to genetics, epigenetics has been explored slowly in the forensic field [11, 12]. DNA methylation is preferred in forensics over other epigenetic modifications (such as changes in chromatin structure or histone modifications) for both in vitro stability and high sensitivity in terms of DNA amounts required. Currently, only a limited number of DNA methylation markers are applied for a few forensic purposes, using technologies that enable the analysis of a small number of such markers. These approaches can be classified as forensic epigenetics, and include DNA methylation profiling for tissue determination [13], age prediction [14], and differentiation between monozygotic twins [15]. The concept of personalized epigenomics, which is already used in medical research [16], has not yet been recognized in the forensic field.

**Fig. 1:**
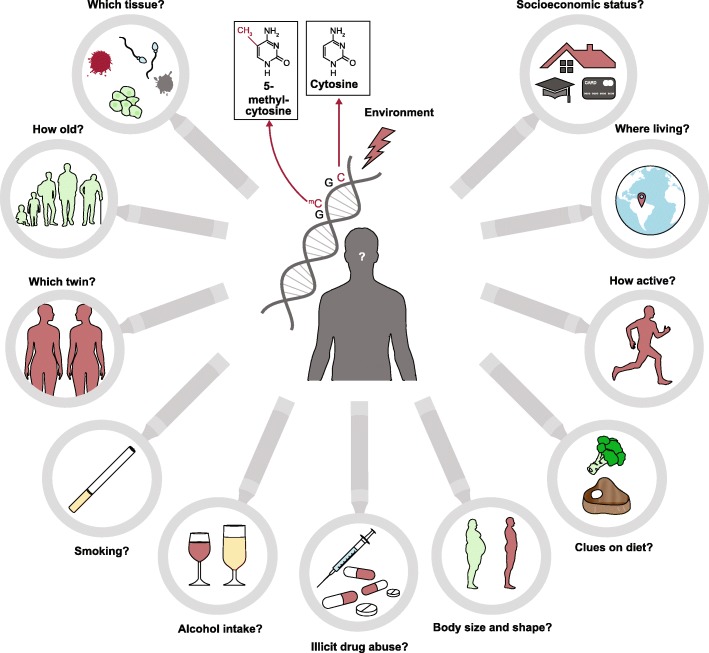
Questions to which forensic epigenomics is envisioned to provide answers in the future

Provided that scientific and technological progress in human epigenomics continues to advance rapidly, we envision the establishment of an “epigenomic fingerprint” [17] from crime scene traces as a promising approach to address various forensically relevant questions that cannot be answered through genetics. We also expect that in the near future novel technologies will be developed to allow the detection of large-scale DNA methylation variation in forensic-type DNA for many more forensic purposes—that is, forensic epigenomics will emerge. These purposes are likely to include the prediction of forensically informative lifestyle and environmental information of an unknown trace donor (Fig. 1) to help further overcome the principle limitation of the current use of DNA in human forensics. Current forensic DNA profiling is completely comparative; that is, it aims to match DNA profiles from crime scene traces with that of known suspects, such as those included in forensic DNA databases [1, 2]. In consequence, perpetrators whose DNA profiles are unknown to the investigators cannot be identified. Together with current emergence of genetic prediction of appearance traits [3] and biogeographic ancestry [4], as well as epigenetic prediction of chronological age [3], epigenomic prediction of lifestyle and environmental exposures will allow further characterization of unknown perpetrators from DNA, which is useful in criminal cases where no DNA profile match has been obtained. If put into practice, such broadened DNA-based intelligence is expected to guide police investigations towards the most likely group of potential suspects.

## Forensic requirements of epigenetic/epigenomic analysis

**Fig. 2:**
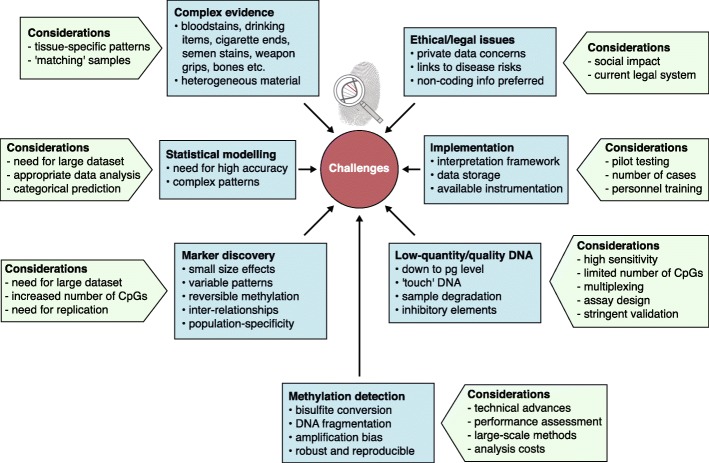
Challenges and considerations in developing and implementing forensic epigenomics. *CpG* cytosine-phosphate-guanine, *pg* picogram

There are several requirements of forensic DNA analysis, which are determined by the low quality and quantity of DNA that is typically available from crime scene traces, which has consequences for the type and number of markers that can be analyzed, and the technology that can be used. These requirements also apply to forensic epigenetic/epigenomic analyses (Fig. 2). Moreover, there are additional technological challenges given the quantitative outcome of epigenetic/epigenomic analysis, in contrast to forensic genetics analysis, which is mostly qualitative.

The limited amount of human biological material available at crime scenes restricts the number of separate DNA tests possible. In consequence, multiplex genotyping methods for the simultaneous analysis of several epigenetic markers at once are required in forensic analysis since single markers typically do not deliver enough forensically useful information. However, currently available technologies for the simultaneous analysis of a large number of epigenetic markers, such as DNA methylation microarrays and whole-genome bisulfite sequencing, are not suitable for forensic trace analysis because of the large input amounts of high-quality DNA they require. At the same time, current epigenetic analysis technologies that are able to deal with low-quality/quantity DNA, such as bisulfite pyrosequencing, methylation quantitative PCR, and EPITYPER®, are limited in their multiplexing capacities (fewer than 20 markers), which are often insufficient to fully address a forensic question of interest [18].

Amounts of DNA obtained from crime scene traces are often low, typically in the picrogram–nanogram range. Therefore, highly sensitive technologies are needed in forensics to allow for reliable detection of DNA variation, including DNA methylation levels. Methods such as methylation SNaPshot with (albeit limited) multiplexing capacity currently have sensitivities down to a few nanograms of DNA input per PCR [13, 19]. However, most current epigenetic methodologies require bisulfite conversion prior to marker analysis; the efficiency of converting unmethylated cytosines into uracils strongly depends on the DNA input. Typically, bisulfite conversion kits require a minimum of 50–200 ng DNA for reliable performance. Reduced DNA input leads to increased technical variation and thus an increased error range of the subsequent DNA methylation analysis. Highly sensitive technologies allowing for simultaneous analysis of large numbers of DNA methylation markers from low-quality/quantity DNA do not yet exist.

Crime scenes traces can consist of different cell types. While cell/tissue-type composition is mostly not restrictive in genetic analysis, it can be challenging in epigenetic analysis. Forensic epigenetic tests have to work equally well in all forensically relevant cell or tissue types or, if that is impossible, need to be tailored to specific tissue types, requiring tissue-type determination prior to epigenetic analysis. Some DNA methylation sites can show substantial differences between different tissues, which needs to be considered when applying previously established predictive marker sets and prediction models to a trace, which can be of a different tissue origin [20, 21]. Even if a large number of epigenetic markers provide tissue-independent information, such as for age prediction [22], reducing the number of markers due to technical constrains in forensic DNA analysis can lead to tissue specificity effects such as in forensic age prediction. Determining forensically relevant tissue types can be achieved via tissue-specific mRNA or microRNA markers [23, 24], which is already established in forensics. If the conclusion of the epigenetic analysis depends on a direct comparison between crime scene material and reference samples, samples from the same tissue type should be used. However, additional challenges in interpretation can be encountered when analyzing heterogeneous forensic-type samples such as whole blood, consisting of different cell types with distinct epigenomes [25, 26].

When it comes to predictive DNA analysis in forensics (and beyond), the accuracy of predicting a trait from DNA, including methylation markers, should be as high as possible. Prediction accuracy should be investigated via different approaches and estimated via different measures in as many test samples as possible. Potential confounding DNA methylation effects [27] caused by a combination of factors such as age or environmental exposures should also be taken into account during interpretation, and properly tested before implementation. However, forensic DNA prediction is generally applied in cases where the police have little or no knowledge of the identity of the trace donor and how to find him/her. Hence, although high prediction accuracies are generally preferred in forensic DNA prediction, including when DNA methylation markers are used, lower accuracies may be accepted given what is known in a specific case and if other information available to the police already has low or unknown accuracies (for example, eyewitness statements).

## Current progress in forensic epigenetics

### What type(s) of cells does the trace contain?

Along with standard DNA profiling, knowledge regarding the cell or tissue type(s) of the crime scene trace can provide crucial information for crime scene reconstruction, since specific tissues indicate particular types of activity. Since epigenetics is involved in cell differentiation and gene expression regulation [28], identifying forensically relevant body fluids is possible using differentially methylated loci. Frumkin et al. [29] first highlighted the potential of epigenetic markers for semen trace determination. Subsequently, several studies have been published using various DNA methylation loci and analysis methods for different forensically relevant tissues [30–33]. Reported genes include *FOXO3* and *EFS* for blood [32, 34], *SLC12A8* and *BCAS4* for saliva [30, 34], *DACT1* and *C12orf12* for semen [31, 35], *LOC404266* and *HOXD9* for vaginal secretion [34], and *SLC26A10* and *LTBP3* for menstrual blood [13]. The reliable epigenetic determination of more complex body fluids such as menstrual blood can be more challenging, mainly due to the combination of different cell types and smaller methylation effects of currently proposed markers [13]. Until now, the only commercial test based on DNA methylation exists for seminal fluid [36, 37]. Non-commercial multiplex test systems targeting several tissues simultaneously have been published recently [13, 38], but currently have not been validated for acceptance in court. Despite the very recent introduction of such tests to criminal casework in some countries (for example, South Korea), future research regarding each marker’s specificity across a wide range of tissues, inter- and intra-individual variation, in vitro stability, gender-, age- and/or ancestry-associated influences, as well as full assessment and validation of the proposed multiplex forensic systems, remains necessary to fully establish practical usefulness in criminal casework.

### How old is the unknown trace donor?

Predicting the lifetime age of an unknown trace donor at time of trace deposition can help police to focus their investigation to find unknown perpetrators [3]. DNA methylation is strongly affected by ageing [22, 39, 40]. Picking-up on genome-wide scans using DNA methylation microarrays [22, 41, 42], forensic (epi)geneticists have started to establish age-associated sites as biomarkers of lifetime/chronological age at genes such as *ELOVL2*, *C1orf132*, *TRIM59*, *FHL2*, *ASPA*, *SCGN*, and *CSNK1* [14, 43–53]. Although an epigenetic age prediction model has been proposed that behaves similarly across human tissues [22], the number of CpGs used (353) is too large for multiplex-based trace analysis with current technologies. When reducing the number of age markers, tissue-specific effects of epigenetic age prediction are evident, so that tissue-specific marker sets and models need to be developed. Forensically motivated age prediction models based on a small number of CpGs have been built mainly for blood [14, 49, 50, 52–54] and less so for saliva [46, 55–57], semen [58], and teeth [44], which deliver age prediction with errors of around ±5 years. However, gender-specific differences and higher errors for old, very young, and diseased individuals (for example, those suffering from age-associated conditions [59]) can be expected [14, 44, 48, 52, 53], which are attributed to the fact that, instead of lifetime age (that is, number of years alive), these epigenetic markers predict biological age (that is, a measure of age-related changes in body function or composition associated with one’s ageing rate). Previous studies [48, 53] have highlighted greater variation in known age versus age predicted with DNA methylation markers for children and elderly people, relative to medium-aged people. This may illustrate the discrepancies between biological and chronological age as detected with epigenetic markers, which are expected to be larger during developmental lifetime and with advanced age compared with medium-aged people. However, most perpetrators of crime are of medium age. Forensically suitable commercial solutions are currently not available despite the increasing interest from police forces worldwide. However, we expect that further research and validation studies will identify robust markers that eventually will be pooled together in multiplex solutions for age estimation from crime scene traces.

### Which twin is the trace donor?

Monozygotic (MZ) twins cannot be individually identified by standard forensic DNA analysis because they share the same DNA profile, which is a drawback for law enforcement. For a service based on ultra-deep whole genome sequencing to detect very rare somatic mutations, a company charges tens of thousands of Euros for a single twin case, which does not guarantee success [60]. Genetically identical MZ twins are sometimes discordant for certain phenotypes [61], indicating epigenetic involvement [6], and several studies have demonstrated that there is considerable epigenetic variation within MZ twin pairs. Although some studies have explored the value of epigenetic profiling in forensically discriminating MZ twins [62, 63], it is not yet fully established whether the observed twin-to-twin differences are twin pair-specific, or might be universal and applicable across twin pairs, as would be preferred. Recently, a first attempt was made to demonstrate the feasibility of differentiating between MZ twins using forensic epigenetics [15]. This study showed that most, but not all, twin-differentiating CpG sites (which were identified using genome-wide screening technologies in reference-type blood DNA) could be replicated by targeted methods that are suitable for forensics in trace-type DNA from bloodstains, highlighting technical challenges [15]. Another key issue that remains unclear concerns the number of epigenetic markers required to achieve statistically sound identification of individual MZ twins, which is an issue as current screening technologies are not suitable for trace analysis. We expect that additional research testing the stability of DNA methylation differences over time and different tissues, technologies, and approaches will determine whether differential DNA methylation is indeed a suitable approach for addressing this forensic question.

## Future perspectives of forensic epigenomics

### Is the unknown trace donor a smoker?

Despite tobacco smoking being widely recognized as having negative health outcomes, a large proportion of the world population still smokes: for example, 19–32% of Europeans [64]. The ability to predict smoking habits from trace DNA would be highly informative in characterizing an unknown trace donor, and thus useful in guiding investigations. Smoking is known to cause DNA damage and telomere shortening [65], and also epigenetic changes, which are caused by effects on DNA methyltransferase expression [66] and DNA methylation patterns [67]. Epigenetic effects of tobacco smoking are also related to cumulative smoke exposure (pack-years) and associated with time since quitting [68–70]. The first epigenome-wide association study (EWAS) in blood aimed to identify differential DNA methylation associated with smoking found a single CpG marker (*F2RL3*) [67]. Following more than 18 additional EWASs in thousands of individuals, various smoking-associated CpGs have been recognized in several genes, including *AHRR* [71–79], *ALPP2* [72–74, 76–78, 80, 81], *GFI1* [73, 74, 76, 82], *GPR15* [74, 75, 81], and *MYO1G* [73, 76, 81, 83]. However, the observed per-site DNA methylation differences are relatively small (usually less than 20%) [84]. While most studies have been performed in blood, smoking-associated CpGs have also been identified in other tissues such as lung [72, 79]. While epigenetic effects are persistent for long periods after smoking cessation, some are reversible [68, 77, 85]. One preliminary attempt to predict smoking habits using epigenetics tested a model combining four CpGs for the ability to differentiate between never (*n* = 120) and former smokers (*n* = 45), achieving a prediction accuracy of area under the curve (AUC) of 0.83 (AUC values range between 0.5 meaning random prediction and 1.0 meaning completely accurate prediction) [86]. Besides further increasing the prediction accuracy by adding more smoking-predictive CpGs, additional challenges should be considered in the future, such as population-specific effects [76, 87]. One important aspect here is the effect of maternal smoking during pregnancy (for example, 10.7% of pregnant American mothers have been reported to smoke [88]), which could cause similar epigenetic changes in the offspring, lasting into puberty and even adulthood. The influence of passive smoking, which could also impact the epigenome, needs to be considered as well in future practical applications of epigenetics to smoking prediction.

### Is the unknown trace donor a drinker?

Alcohol intake highly varies between countries and individuals (more than one-fifth of European adults experience weekly “binge” drinking [89]), and predicting drinking habits can be useful for investigative purposes. Forensic toxicological tests for alcohol metabolite detection exist for blood, urine, and hair, but do not allow inferences regarding regular drinking habits (i.e., how often and how much alcohol is consumed). Due to both genetic [90] and environmental factors [91], differential DNA methylation is evident in regular alcohol consumers versus non-drinkers. A significant increase in global blood methylation has been observed in chronic alcoholics [92], while genes such as the dopamine transporter [93] have been shown to be differentially methylated in alcohol-dependent individuals, although this finding has not yet been replicated in other studies [94]. The first EWAS for alcohol dependency revealed numerous epigenetic markers associated with alcohol metabolism [95], the majority of which (1702 CpGs, *p* < 0.005) were hypomethylated in alcoholics versus non-drinkers (<17% difference). This finding, however, contradicts alcohol-associated hypermethylated genomes reported elsewhere [92, 96]. In another study, 865 hypomethylated and 716 hypermethylated CpGs were identified [97]. In the largest meta-analysis available, five CpGs were highlighted to explain a substantial proportion (5.2–15%) of interindividual variance in alcohol consumption and were thus proposed as biomarkers for heavy alcohol drinking [98]. A preliminary prediction study achieved AUC > 0.90 based on 144 CpGs [98], a number that from a forensic standpoint is challengingly high due to limited crime scene material and current method capabilities. More candidate markers have been revealed recently, but with effects as small as 1–5% [99]. Alcohol-dependent epigenetic signatures are partly reversible upon abstinence [99] and, as with smoking, prenatal maternal alcohol intake (which occurs in 9.8% of pregnancies worldwide, 2017 [100]) alters gene-specific methylation in placental cord blood [101], and this could potentially lead to false-positive predictions. We expect that future research will identify robust markers to be included in a forensically suitable prediction tool.

### Is the unknown trace donor an illicit drug user?

Illicit drug use is prevalent in adults, ranging from 1 to 41% depending on the country [102], and is therefore relevant in characterizing unknown trace donors. Commonly used drugs include cannabis, cocaine, and amphetamines. Depending on the country’s legal framework, thousands of drug-related offences occur annually [102]. Similarly to alcohol, forensic toxicological tests are in widespread use; however, they do not provide information on history and habitual use (possibly except for hair analysis). Most studies on drug-induced epigenetic changes have been performed in animal models [103, 104], mainly focusing on chromatin structure and histone modifications [105]. Drug-induced DNA methylation changes have been recently investigated in animal brain regions and neural cells [106]. Global methylation levels were not different in mouse brain and liver following chronic heroin or cocaine treatment [104], but in human brain results were contradictory following methamphetamine dependence [107]. Applying candidate gene approaches, only cannabis and opioid epigenetic effects have been studied in blood thus far. Cannabis-dependent individuals demonstrated altered blood *CB1* methylation, which is also detected in cigarette smokers [108]. Almost 200 heroin addicts showed altered blood *OPRM1* methylation, but per-site changes were small (<4%) [109] and showed population differences [110]. These so-far small methylation differences indicate that larger numbers of individuals need to be included in association studies; however, due to expected difficulties in performing such studies with controlled drug use by study participants, this research question remains in its infancy. Future experiments are also needed to determine whether epigenetic differences are anticipated only in the brain (where the drugs’ effects occur), or whether these are also detectable in forensically more relevant tissues, such as blood. Finally, drug dose-dependent and reversible effects are also expected.

### Are there any diet indications for the unknown trace donor?

Predicting an unknown individual’s diet can be of forensic relevance, when special diets are followed (e.g., vegetarian) or special foods are consumed that can potentially be linked with a particular characteristic, such as geographical location, tradition, and religion. Individual staple food comprises various major components such as fruits, vegetables, meat, and fish. Eventually, dietary differences are translated into different intakes of macro- and micronutrients, including carbohydrates, protein, fat, vitamins, and minerals. Nutritional epigenomics is a relatively new, but rapidly growing, research field [111]. Micronutrients including folate and B vitamins seem to play a key role as secondary methyl donors [112]. One of the first epigenome-wide studies demonstrated that prenatal exposure to famine causes lifelong methylation changes [113]. A cross-generational study identified 134 “nutrition-sensitive” regions, implicated with impairments in attention/cognition [114]. Comparing different eating patterns, European vegetarians were found to have approximately 40% decreased *MnSOD* buccal methylation compared with omnivores [115], while in a smaller-scale study, plasma homocysteine levels showed a significant correlation with global blood DNA methylation in vegetarians [116]. Looking at specific macronutrients, mercury exposure via fish consumption causes *SEPP1* hypomethylation [117], and daily intake of roasted meat alters *p16* methylation in oesophageal tissue cells [118]. Dietary folate from fortified foods has also been positively associated with LINE-1 blood methylation [119]. Overall, diet involves complex, variable patterns and processes. It is unknown whether inter-relationships between different macro- and micronutrients exist and how unique the observed epigenomic effects are to a specific food type or nutrient. Nevertheless, we envision that future large-scale epigenomic analysis of different diet groups, such as vegetarians versus non-vegetarians, may allow the construction of prediction models that have the potential to be used in forensic applications.

### Is the unknown trace donor physically active?

Information on an unknown person’s physical activity levels might provide insights on their body structure and appearance, which is relevant when describing an unknown trace donor. Physical exercise can impact the epigenome [120] and regulate gene expression [121]. It is also involved in gene–environment interactions that reduce genetic effects on individuals’ body mass index (BMI) [122]. Whereas cross-sectional and case–control studies revealed no significant correlation between physical activity and global blood methylation [123], LINE-1 methylation was increased in women maintaining higher physical activity over a long period of time [124]. Exercise-related epigenetic effects were also stronger in elderly populations [125], diseased individuals (*L3MBTL1*) [126], and in tissues such as fat (*TCF7L2*) [127] and skeletal muscle (*KCNQ1*) [128]. Being physically fitter or exercising regularly correlates with lower cancer gene methylation in saliva [129]. When testing the effects of regular moderate exercise on inflammatory response via epigenetic changes in blood, there was no effect regarding the *IL-6* [130] and *p15* [131] genes, but a reduced age-dependent *ASC* blood methylation was observed [131]. Thus far the effects of long-term, rather than acute, exercise have been studied, so it is unknown when methylation changes are established and become detectable in relation to timing of exercise. This research is still at early stages and ongoing, but future large-scale experiments including controlled exercise regimes for study participants have the potential to identify distinct exercise-related epigenetic differences. Depending on the outcomes, a forensic tool may be developed to predict whether an unknown trace donor is physically active or not.

### What is the body size/shape of the unknown trace donor?

While predicting categorical externally visible characteristics such as eye and hair color is already established [132], predicting dimension-based features, such as body height, is challenging due to their continuous quantitative nature. Although the genetic component of body height is large [133, 134], environmental factors explain about 20% of height variation. Due to the immense genetic complexity of height, despite very large genome-wide association studies (more than 250,000 subjects, the identified SNPs do not explain more than 27.4% of the phenotypic variation [135, 136]. For BMI, however, this figure is just 2.7% [137]. There is increasing evidence that epigenetic variation might play a role in shaping body height [138] and BMI [139]. The first study in humans identified that 83% of height-associated genes contain promoter CpG islands linked with gene regulation, half of which had significant DNA hypermethylation modules [138]. While there is currently no published EWAS for height, studies in other species such as *Arabidopsis thaliana* [140], ants [141], and sheep [142] have identified height-associated methylation in body-size-related genes. In the case of BMI, where EWASs have been carried out for humans [139], birth-weight discordant twins did not show significantly different epigenome-wide profiles [143], but three CpGs (in the gene *HIF3A*) were found to be significantly associated with BMI in a larger cohort of unrelated individuals [139]. For every 10% methylation increase of cg22891070, BMI was approximately 3% higher [139]; however, these effects were not replicated in adolescents [144]. Following a comprehensive scan of about four million CpGs, four BMI-associated variably methylated regions (*PM20D1*, *MMP9*, *PRKG1*, and *RFC5*) were discovered [16]. In the largest meta-analysis to date, the BMI-associated DNA methylations levels for 187 loci were successfully replicated in multiple tissues and ethnic groups [145]. In another study in CD4+ T cells, eight additional BMI- and waist circumference-related CpGs were identified [146]. We envision that currently identified CpGs, together with future outcomes from large-scale epigenetic studies, may form a suitable marker pool for a future forensic tool to predict a person’s body height and weight, which in combination with physical activity information can create a more detailed picture of the physique of an unknown individual.

### In which geographic region does the unknown trace donor live?

Predicting biogeographic ancestry via small sets of genetic markers is feasible in current forensic testing, at least at the continental level [4]. However, the geographic regions where the ancestors of a person originate from is not necessarily the same as the region where the individual lives (residency), especially in the current age of globalization [147]. Currently, residency can be inferred via isotope analysis [148], but this is unsuitable to crime scene traces. Genetic geographic population substructure, which is the basis of genetic ancestry inference, is caused by human migration and positive selection via local genetic adaptation to environmental factors, which occur over large periods of time involving multiple generations. By contrast, epigenetic geographic population substructure influenced by local environmental factors is produced much more quickly, and within a person’s lifetime. Giuliani et al. proposed that the factors influencing spatial epigenetic variation are mainly nutrients, UVA exposure, and pathogens [149]. Distinct epigenetic changes due to chronic sun exposure have been found in human skin (*KRT75*) [150], while environmental chemicals such as cadmium exposure via soil in Thai populations [151] and phthalate exposure via household products in the USA [152] affect gene-specific DNA methylation. Apart from metals and organic pollutants [153], others such as water contaminants and airborne pollution could have similar effects. Lifetime exposure to undesired disinfection products formed during water treatment caused methylation differences in 140 CpGs in Spanish individuals [154], while mitochondrial DNA (mtDNA) methylation was altered in Italian steel workers due to their high exposure to metal-rich particulate matter [155]. Nevertheless, these mtDNA methylation changes are considered minute, since overall mtDNA methylation seems to be less than 6% [156]. Overall, we regard it as likely that, besides biogeographic ancestry information from genetic markers, additional residence information via epigenetic profiling will become available in the near future with additional benefits for investigative use.

### Are there hints about the socioeconomic status of the unknown trace donor?

Socioeconomic status (SES) is often measured as a combination of education, occupation, income, and marital status, thus viewed as a continuous variable; it is conceptualized as the social class of an individual, associated with behavioral features and disease risks [157–159]. While complex and highly variable, information about the SES of an unknown trace donor could help police target their investigations. Together with genetics and physical environment, social factors also impact on epigenetic variation [160]. Well-defined epigenetic patterns have been linked to both childhood and adulthood socioeconomic environment [161]. Early-life SES was found to be associated with altered methylation in three CpG sites in blood, but the methylation effects were low (<5%) [162]. Following candidate gene approaches in multiple populations, SES-associated methylation was also reported in stress-related (*AVP*, *FKBP5*, *OXTR*) and inflammation-related (*CCL1, CD1D*, *NFATC1*) genes [163, 164]. In another study, low-SES was also linked with altered methylation of the serotonin transporter gene [165, 166]. Looking at global DNA methylation and job status in particular, manual workers demonstrated 24% global hypomethylation compared with non-manual workers [167]. Various SES-associated factors, including family income at birth [168], adult education [168], maternal education [169], parenting [170], and status of single parent family [168], have all been linked with altered methylation at specific genomic locations. While this research is still ongoing, following comprehensive characterization of SES-associated effects it might be possible in the near future to be able to translate an individual’s epigenome into clues regarding their educational, occupational, and marital status; however, distinct predictions might be unlikely.

## Ethical and societal issues of forensic epigenomics

Predicting lifestyle and environmental factors of unknown forensic trace donors via epigenomic profiling may raise ethical and social issues and concerns and, depending on a country’s legal framework, may require legislation regulations before being put into forensic practice. DNA-based prediction of appearance traits and biogeographic ancestry for investigative purposes (referred to as forensic DNA phenotyping (FDP) [3]) has already given rise to such issues, and opinions between expert scientists vary [2, 3, 171–173]. To date only a few European countries allow FDP in forensic practice, such as the Netherlands, UK, and France [3, 174], as well as some states in the USA. Notably, this situation is currently changing, as policy makers in some countries, such as Germany and Switzerland, are considering allowing appearance and ancestry DNA testing for investigative forensic use. In other European countries, including Spain, Sweden, and Poland, FDP can be legally practiced as legal restrictions apply only to genetic markers used in forensic DNA databases.

It could be argued that ethical concerns regarding privacy protection and the right not-to-know (and thus not wanting others to know) are less pressing regarding the genetic prediction of obvious appearance traits because their external visibility cannot be considered private. This reasoning may also apply to epigenetic prediction of those lifestyle factors that are obviously visible, such as tobacco smoking, or those that are generally viewed positively, such as physical activity. However, lifestyle factors with epigenetic signatures that are generally viewed negatively may be hidden by individuals from public exposure, which makes privacy issues more of a concern. Nevertheless, as previously discussed among ethics experts, some unhealthy lifestyle factors, such as smoking, are considered non-sensitive behavioral traits, while others, such as alcohol drinking, belong to a middle category of “somewhat but not too sensitive” traits [175], in contrast to those, such as use of illicit drugs, that are legally forbidden. Some lifestyle and environmental factors represent known risk factors for diseases, where the right not-to-know can apply (regarding the disease risk); however, based on current knowledge, none of these factors provides a direct link with sensitive medical information, which should make their epigenetic prediction less problematic.

In contrast to genetic data in forensic DNA profiling, and as with genetic data from appearance and ancestry prediction, epigenetic/epigenomic data from lifestyle prediction are not stored in central forensic databases. Only the trait information (that is, the probability of displaying a certain trait or being influenced by a certain lifestyle factor), but no actual genetic/epigenetic data, should be communicated to the police for use in investigations. Ethical and societal issues of probabilistic epigenomic lifestyle prediction should be discussed among interdisciplinary groups of experts, including representatives with (epi)genetics, forensic, ethics, social, and law expertise, before practical applications can be considered.

## Conclusions

Epigenetic applications in forensics are relatively new and currently limited, but we expect a rapid development towards forensic epigenomics in the near future. While today only three forensically relevant issues are investigated via epigenetics, we envision an expansion towards forensic epigenomics for addressing at least some of the investigative questions proposed here. The extent to which such broadening of forensic epigenetics into forensic epigenomics will happen will depend on several factors. First, further scientific progress in cataloguing and understanding epigenetic signatures of lifestyle and environmental factors. Second, identifying epigenetic markers and building/validating statistical models for accurate epigenetic lifestyle prediction. Third, technical progress in simultaneous analysis of large numbers of epigenetic markers from low-quality/quantity DNA (potentially through new technologies such as Oxford Nanopore sequencing) and developing/forensically validating sensitive multiplex analysis assays. Finally, ethical and societal discussions on the benefit versus risk of using such human epigenetic data in forensic practice with consequent legal implementations if deemed necessary. If it is eventually applied in forensic practice, epigenomic prediction of lifestyle/environmental factors will enhance DNA investigative intelligence by complementing genetic prediction of appearance and biogeographic ancestry and epigenetic prediction of lifetime age, all aiming to guide police investigations towards finding unknown perpetrators of crime who are unidentifiable with standard forensic DNA profiling.

## Abbreviations

AUC: Area under the curve
BMI: Body mass index
CpG: Cytosine-phosphate-guanine
EWAS: Epigenome-wide association study
FDP: Forensic DNA phenotyping
mtDNA: Mitochondrial DNA
MZ: Monozygotic
SES: Socioeconomic status
